# Carbonic Anhydrases and Their Biotechnological Applications

**DOI:** 10.3390/biom3030553

**Published:** 2013-08-19

**Authors:** Christopher D. Boone, Andrew Habibzadegan, Sonika Gill, Robert McKenna

**Affiliations:** Biochemistry & Molecular Biology, University of Florida, P.O. Box 100245, Gainesville, FL 32610, USA; E-Mails: cdboone@ufl.edu (C.D.B.); ahabibzadegan@ufl.edu (A.H.); sonika22@ufl.edu (S.G.)

**Keywords:** carbonic anhydrase, artificial lungs, biosensor, carbon sequestration

## Abstract

The carbonic anhydrases (CAs) are mostly zinc-containing metalloenzymes which catalyze the reversible hydration/dehydration of carbon dioxide/bicarbonate. The CAs have been extensively studied because of their broad physiological importance in all kingdoms of life and clinical relevance as drug targets. In particular, human CA isoform II (HCA II) has a catalytic efficiency of 10^8^ M^−1^ s^−1^, approaching the diffusion limit. The high catalytic rate, relatively simple procedure of expression and purification, relative stability and extensive biophysical studies of HCA II has made it an exciting candidate to be incorporated into various biomedical applications such as artificial lungs, biosensors and CO_2_ sequestration systems, among others. This review highlights the current state of these applications, lists their advantages and limitations, and discusses their future development.

## 1. Introduction

Three analogous families of carbonic anhydrases (CA) exist within nature: α-CAs (predominant within animals), β-CAs (predominant within plants), and the γ-CAs (predominant within Archaea) [1,2,3,4,5]. In total, there are 15 human α-CA isoforms, all of which differ in their catalytic rates, inhibitor sensitivity and selectivity, cellular localization and tissue distribution [1,6,7]. The 12 catalytically active human isoforms (HCAI–VA, VB–VII, IX, XII–XIV) exhibit a wide range of catalytic efficiencies (k_cat_/K_M_ = 10^3^−10^8^ M^−1^ s^−1^). The acatalytic human CA-related proteins (HCA-RPs VIII, X and XI) are inactive due to the evolutionarily loss of one or more of the zinc-coordinating histidine residues, leading to loss of the zinc metal from the active site [1,8].

CAs are involved in various physiological roles fluid secretion, acid/base balance and thus pH regulation, gluconeogenesis, ureagenesis, gastric acid production, and transport of CO_2_ from tissues to the lungs (in the form of bicarbonate) through blood [4,9,10]. CO_2_ released as a part of respiration by tissues is not very soluble in blood and thus, in order to be transported, is converted to HCO_3_^−^ by HCA II. Furthermore, the role of CA in diseases such as glaucoma has long been known. Over secretion of aqueous humor in the eye causes increased intra-ocular pressures consequently leading to a condition called glaucoma. Reduction in CA activity decreases the secretion of HCO_3_^−^ and aqueous humor, thereby reducing the pressure [1,7,11,12].

The best characterized of these enzymes is HCA II, found within the cytosol of many cells and organs [9,13,14,15]. Known to possess a remarkably high catalytic efficiency, with a k_cat_ of 1.4 × 10^6^ s^−1^ and a k_cat_/K_M_ of 1.5 × 10^8^ M^−1^s^−1^ [6,16,17], HCA II aids in the conversion of water and carbon dioxide into bicarbonate and a proton through a two-step ping pong mechanism:

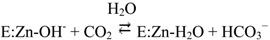
(reaction 1)

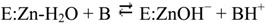
(reaction 2)


In the hydration direction shown, in the first step the zinc-bound hydroxide acts as a nucleophile, attacking the carbon dioxide and ultimately forming bicarbonate. This leads to a water molecule bound to the zinc (reaction 1). The second step (reaction 2) regenerates the zinc-bound hydroxide through a proton transfer mechanism via His64 in HCA II [18] to solvent, B [19,20,21,22].

This review will discuss the current state of utilizing HCA II in the biomedical field to aid in the development of an artificial lung system, as a biosensor for trace elements in complex media and in CO_2_ sequestration among confined spaces, followed by a short discussion on other systems. HCA II is a particularly attractive candidate for these applications because of its relatively high stability [23], ability to be expressed in large quantities from *E. coli* [24], and the relatively easy purification from either affinity or conventional chromatography [25,26]. Additionally, the available high-resolution X-ray and medium neutron crystallographic structures of HCA II [27,28,29,30] allow for the rational engineering via site-directed mutagenesis for enhanced catalytic activity [31,32] and stability [33,34,35] for various industrial [36,37] and medical [1,38,39,40] applications.

## 2. Artificial Lungs

One of the current prevailing health problems within the United States is respiratory failure [41]. A common treatment for this condition is the utilization of mechanical ventilators [42]. Unfortunately, these ventilators can create many problems for patients who are treated with them, including decreased lung efficacy because of over-pressurized or over-distended lung tissue [43]. An artificial lung is a device capable of assisting with respiration without input from the lungs. This technology could supersede ventilators in treating respiratory failure. There are still many challenges facing this new technology, however, that must be dealt with before artificial lungs replace mechanical ventilators [44].

As of now, the main issue preventing effective artificial lungs concerns inadequate transfer of CO_2_ per square inch across the polymetric hollow fiber membranes (HFM) present within this technology. Currently, a 1–2 m^2^ surface area is required to sufficiently transfer CO_2_ through the membrane [45,46,47,48]. A surface area of this size lacks the practicalities of functioning effectively within the human body [49,50]. One way that has been effective at increasing the transfer of CO_2_ lies in immobilizing CA onto the HFM. The enzyme, dissolved in a phosphate buffer, is added to the surface of the HFM. Cyanogen bromide within acetonitrile activates the HFM, allowing covalent bonds to form between CA and the HFM. Transfer rates of CO_2_ measured with CA treated compared to untreated HFM show a 75% higher rate of CO_2_ removal rate present in with the treated HFM. This finding indicates the possibilities for smaller artificial lungs to be engineered with CA incorporation, which could function effectively within the human body [51].

Another method that has been shown to also increase CO_2_ transfer across the HFM of artificial lungs relies on impeller devices that increase the rate of blood mixing. Unfortunately, this method cannot be combined with the CA method at this time. When these two methods were combined, the shear forces of the impeller device denatured CA, leading to a loss of enzyme function. This creates the need for a more stable form of CA that will not be denatured by the shear forces. If such a stable CA variant can be engineered, these methods might be combined, which could lead to smaller more efficient artificial lungs [52].

## 3. Biosensors

Quantification of trace analytes in complex media containing chemically similar molecules is lacking in many traditional chemical systems. As such, the development of sensors based on biological molecules, termed biosensors, can achieve such specificity and sensitivity [53]. The high affinity of HCA II for zinc (4 pM) [54] has been used to quantify trace amounts of zinc in sea and waste water [55] for concerns over toxicity to certain plants, invertebrates and fish [56,57]. Optimally, this biosensor would operate along the sea bed and relay a fluorescence signal up to the ocean surface that is released upon binding of a strong inhibitor, dansylamide, upon binding of zinc in the active site of apo-CA [58]. However, the slow dissociation rate of zinc from the CA active site (t_1/2_ ≈ 90 days [54]) limits the reusability and efficiency of the system. The relative abundance of natural zinc in the environment compared to that of the binding affinity also limits the production of apo-HCA II [23]. As such, an HCA II variant (E117Q) that contained both a lowered binding affinity (nM) and a much faster dissociation time (t_1/2_ ≈ 3 sec) for zinc was developed to circumvent these limitations [59]. Other studies have aimed to improve the fluorescence signaling upon zinc binding in the active site of HCA II via incorpororation of the H36C variant, which then selectively labeled with a thiol-reactive fluorophore [60] that would interact upon ligation of an inhibitor, azosulfonamide, acting as a fluorescence acceptor [61].

Other metals that bind to HCA II consist primarily of transition metals in the +2 oxidation state, which include: Cd^2+^, Co^2+^, Cu^2+^, Hg^2+^, Fe^2+^, Mn^2+^, Ni^2+^, Pb^2+^ and In^3+^ [53,54,60,62]. However, only the binding affinity of HCA II for Cu^2+^ and Hg^2+^ is greater than that of Zn^2+^ [54], so variants with a lowered binding affinity for these metals would have to be developed before detection of other metals is feasible. Sulfonamide inhibitors, however, do not bind tightly to CA with metals other than Zn^2+^ or Co^2+^ bound in the active site [63], promoting the need for a novel development of metal ligation. This limitation can been superseded because several of these divalent ions (Cu^2+^, Co^2+^ and Ni^2+^) exhibit weak d-d absorbance bands in the visible regions that can be directly measured via fluorescence energy transfer lifetimes [60]. This can be extended to the other metals that do not exhibit d-d absorbance bands, (Hg^2+^ and Cd^2+^) since the binding of these metals to apo-CA causes a quenching of the fluorescence of an active site fluorophore [64]. Since biologically prevalent divalent metals such as Mg^2+^ and Ca^2+^ do not bind to CA and interfere with the assay, biosensors employed in biomedical applications are especially useful [65,66,67,68,69,70].

## 4. CO_2_ Sequestration

Elevated CO_2_ levels in the human body have detrimental effects ranging from impaired judgment to death. CO_2_ control is important in confined spaces where there is little buffering ability to absorb this gas, such as spacecraft or submarines. These life support systems employ a small amount of CA dissolved in thin aqueous buffered films and compressed between porous polypropylene membranes [71]. The concentrations of CO_2_ commonly experienced in these systems (~0.1% v/v) are ideal for selective capture by CA. Analysis of the produced (scrubbed) gas shows that the CA-containing setup selectively lets N_2_ and O_2_ through, with ratios of 1400:1 and 900:1, respectively [71,72]. The relatively low concentration of CO_2_ readily dissolves in the thin layer of enzyme containing buffer and across the membrane, where it is removed via vacuum or carrier gas. Engineered CA-based bioreactors outperformed chemical methods using diethylamine solutions, with much higher selectivity, 400:1 and 300:1 for N_2_ and O_2_, respectively [72]. In addition, the presence of CA increased CO_2_ transport across the polypropylene membrane by ~70% [71]. Another benefit of these CA-bioreactor systems is that they are very efficient at ambient pressures and temperatures [72], improving overall cost-efficiency. However, the longevity of the systems has raised concerns as the need to keep the membranes wet, or at least humid, will add cost and operational difficulties to their practical use.

## 5. Pharmalogical Considerations

CAs have been employed in CO_2_-responsive cationic hydrogels in antidote delivery to treat analgesic overdose without losing therapeutic levels of drug [73]. Alternate medicines, such as opioids, have very potent analgesic effects but overdoses can cause respiratory hypoventilation, which leads to increased CO_2_ and decreased O_2_ levels in the body ultimately leading to an acidosis-induced death. The CA treatment involves a feedback-regulated antidote delivery system that responds to high CO_2_ levels or decrease in pH [73]. The cationic hydrogel is based on N,N-dimethylaminoethyl methacrylate (DMAEMA) polymers that have modified to have a pK_a_ ~ 7.5, which makes it an adequate blood pH monitor. Incorporation of CA as a CO_2_ sensor improved the efficiency of these antidote-delivery systems [73]. Other hydrogels have been designed with a switchable co-block polymer can undergo a transition change from gel to sol upon exposure to CO_2_ [74]. This study demonstrates that stimuli-triggered drug delivery could be incorporated with CAs utilizing CO_2_, bicarbonate or pH changes as signaling molecules.

High-resolution X-ray [75,76] and neutron structures [29] of HCA II in complex with acetazolamide (Diamox), a tight binding inhibitor used in the treatment of glaucoma [12,77], has accelerated research into structural-based rational design of an isozyme specific inhibitor. Current research interest includes specific inhibition of HCA IX, which has been shown to be overexpressed in a wide array of cancer cell lines [11,39,78,79,80]. In short, tumor cells proliferate in acidic environments which could be presumably due to the catalytic activity of HCA IX on the cell surface. Selective inhibition of HCA IX could provide a means for targeted tumor eradication. A suitable drug candidate has not been discovered as of yet, but an impressive library of inhibitors designed from different functional groups with varying binding affinities and specificities for various CA isoforms has been steadily growing with entries and has been summarized elsewhere [2,10,38,40,81,82].

## 6. Blood Substitutes

A continual source of blood is required for use in trauma injuries or major surgeries, and, as natural blood is often in limited supply, there has been progress in the development of blood substitutes which primarily consist of 4–5 cross-linked stroma-free hemoglobin (polySFHb) molecules [83]. The major drawback of these substitutes, however, was the inadequate CO_2_ removal rates. Increased CO_2_ levels in the body leads to acidosis, and if left untreated will end up in coma and death [9]. Incorporation of catalase (CAT), superoxide dismutase (SOD) and CA to the PolySFHb substitute (PolySFHb-SOD-CAT-CA) was introduced to overcome this limitation with encouraging activity [84]. Blood substitutes have also been shown to be advantageous over transfused whole blood in that they can be sterilized, stored for long periods and contains no blood antigens [83].

## 7. Conclusions

The various aforementioned biotechnological aspects of the different CA-associated systems emphasize the usefulness of this enzyme. The advancement of fast and cost-effective genome sequencing, molecular biology techniques that boost overexpression of protein and direct evolution techniques that can select for highly active and stable CAs provide an optimistic view as to the advancement in the efficiency and selectivity in current systems. It is also likely that further developments in these fields will lead to novel biomedical applications of CAs.
